# Practical guide to cardiopulmonary exercise testing in adults

**DOI:** 10.1186/s12931-021-01895-6

**Published:** 2022-01-12

**Authors:** Thomas Glaab, Christian Taube

**Affiliations:** 1 Department of Pulmonary Medicine, Mainz University Hospital, Mainz, Germany; 2 MVZ Urdenbacher Allee, Düsseldorf, Germany; 3 Department of Pulmonary Medicine, University Medical Center Essen-Ruhrlandklinik, Essen, Germany

**Keywords:** Exercise limitation, Dyspnea, Ventilatory inefficiency, Cardiovascular disease, 9-Panel plot, Pulmonary hypertension, COPD, Interstitial lung disease

## Abstract

Unexplained exertional dyspnoea or fatigue can arise from a number of underlying disorders and shows only a weak correlation with resting functional or imaging tests. Noninvasive cardiopulmonary exercise testing (CPET) offers a unique, but still under-utilised and unrecognised, opportunity to study cardiopulmonary and metabolic changes simultaneously. CPET can distinguish between a normal and an abnormal exercise response and usually identifies which of multiple pathophysiological conditions alone or in combination is the leading cause of exercise intolerance. Therefore, it improves diagnostic accuracy and patient health care by directing more targeted diagnostics and facilitating treatment decisions. Consequently, CPET should be one of the early tests used to assess exercise intolerance. However, this test requires specific knowledge and there is still a major information gap for those physicians primarily interested in learning how to systematically analyse and interpret CPET findings. This article describes the underlying principles of exercise physiology and provides a practical guide to performing CPET and interpreting the results in adults.

**Supplementary Information:**

The online version contains supplementary material available at 10.1186/s12931-021-01895-6.

## Background

“It is likely that no test in medicine is as informative and cost-effective as cardiopulmonary exercise testing for distinguishing among the broad spectrum of disorders causing symptoms of exercise intolerance. Without it, the evaluation of patients with exercise intolerance may be too narrowly focused by the physician’s particular subspecialty.” (Preface Wasserman & Whipp’s Principles of Exercise Testing and Interpretation, 6th. edn. [1]).

Cardiopulmonary exercise testing (CPET) is a maximal exercise test with concomitant gas exchange analysis that provides an integrative and comprehensive assessment of physiologic responses to exercise and cardiorespiratory fitness. In contrast to exercise ECG, the direct noninvasive determination of minute ventilation, heart rate and expired gases analysis (oxygen uptake and carbon dioxide output) at rest and during exercise provides accurate and reproducible data on the interaction of ventilation, gas exchange, and cardiovascular and musculoskeletal function, and enables determination of deviations from normal.

Use of CPET detects abnormalities in the functional capacity of these organ systems that are amplified or are only present during exercise (e.g., coronary arterial disease [CAD], right-to-left shunt [R-L shunt]) and helps to define the pathophysiology of exercise limitation. It is important to note that patient report of symptoms or stated levels of exercise intolerance correlate only modestly with resting functional and imaging tests [1–3]. As a result, CPET can be particularly valuable in identifying the source of exercise intolerance, monitoring disease progression, evaluating treatment responsiveness and providing information about prognosis.

There are many indications for CPET. The most common of these include [1, 4–12]:

- determining the cause(s) and severity of exertional dyspnoea, exercise intolerance or exercise-induced hypoxaemia;
- assessing exercise capacity and estimating prognosis in various disease states (including chronic heart failure);
- assessing perisurgical and postsurgical complication risk (e.g., for thoracic, heart and visceral surgery; surgical and bronchoscopic lung volume reduction);
- early detection and risk stratification of cardiovascular, pulmonary vascular and lung diseases, and musculoskeletal disorders;
- measuring the response to treatment (e.g., drugs, rehabilitation);
- guiding and monitoring individual physical training in rehabilitation (e.g., cardiac, pulmonary), and in preventive and sports medicine;
- evaluating the limitations/impairments of individual maximum and continuous exercise capacity in occupational medicine.

The absolute contraindications to CPET are consistent with those of exercise ECG [1, 3, 5, 8]. Recommendations for CPET during endemic, epidemic and pandemic health conditions such as COVID-19 have recently been issued [13, 14].

The objective of this practical introduction is to describe the basic principles of exercise physiology and provide an easy-to-follow approach for those primarily interested in learning how to conduct, analyse and interpret CPET in their clinical practice. For further information, reference is given to the literature [3, 5–7, 10, 11, 15–20] and the updated reference work [1].

### Understanding exercise physiology

The transport of oxygen to body tissues depends largely on cardiac output, haemoglobin (Hb) concentration, Hb oxygen saturation, arterial vascular tone and the density of the capillary network.

**Fig. 1:**
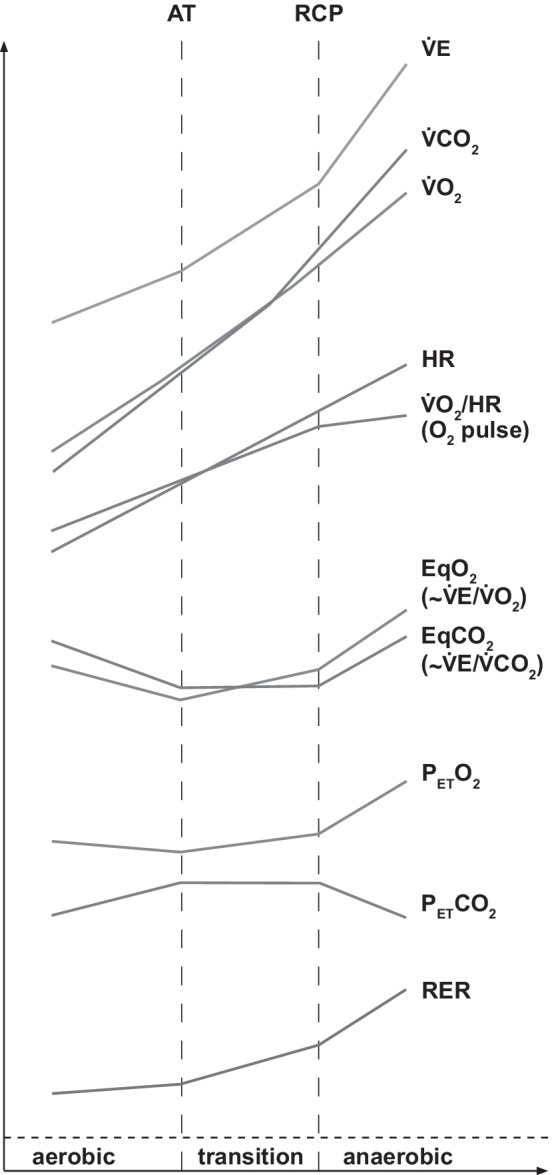
Principles of exercise physiology. The characteristic changes in key variables of ventilation, cardiocirculation and pulmonary gas exchange during progressive exercise are shown. Anaerobic threshold (AT) documents the transition to mixed aerobic-anaerobic metabolism, respiratory compensation point (RCP) documents the transition to predominant anaerobic metabolism. A more detailed description of Fig. 1 can be found in Additional file 1 (see Supplementary Information)

A basic working knowledge of exercise (patho)physiology and gas exchange is fundamental to understanding the pathophysiology of exercise intolerance and to the proper analysis and interpretation of CPET. Figure 1 illustrates characteristic alterations of key physiological parameters as exercise work rate is increased.

### Ventilation/perfusion mismatching

The ratio of ventilation (V) to perfusion (Q) is decisive for the quality of the gas exchange in the lungs. Pronounced ventilation/perfusion mismatch (V/Q) occurs in pulmonary disease, pulmonary vascular disorders and heart failure [21, 22]. Therefore, gas exchange measurements are central to the understanding of the pathophysiology of exercise limitation.

Due to gravity, at rest there is a small gradient in ventilation and a much more pronounced gradient in perfusion from the apex to the bottom of the lung in the upright position (e.g., V/Q drops from the apex [high V/Q] to the bottom) and renders gas exchange ineffective. During exercise, these V/Q heterogeneities diminish, because the upper lung segments are now well ventilated and perfused through deep inhalation, vascular dilation and recruitment of previously closed capillaries. The result is an enlarged gas exchange area. In principle, two kinds of ventilation/perfusion disturbances reflecting true ventilatory inefficiency can be distinguished, and these often overlap:

1. low V/Q regions (incomplete ventilation disorder; shunt effect). The ventilation to perfusion ratio decreases in subventilated but normally perfused alveoli (relative hypoventilation). Examples include chronic obstructive lung diseases (COPD) and restrictive lung diseases (pulmonary fibrosis). As a net effect, hypoxaemia occurs as a result of venous admixture, which cannot be compensated for by hyperventilation, resulting in an increase of P(A-a) O_2_. Elevated PaCO_2_ is usually prevented by hyperventilation in other lung areas. The different effects on PaO_2_ and PaCO_2_ are attributable to the different dissociation curves of O_2_ (sigmoidal form of the O_2_ dissociation curve) and CO_2_ (linear form of the CO_2_ dissociation curve). Hypoxic pulmonary vasoconstriction is one mechanism to redistribute perfusion to better oxygenated regions and thus limit the extent of hypoxaemia.
2. high V/Q regions (incomplete distribution disorder; increased dead-space ventilation [VD/VT↑]). In normally ventilated but poorly perfused alveoli (relative hyperventilation), the V/Q ratio increases (dead-space effect). Examples include pulmonary emphysema (compression effects due to hyperinflation, reduced capillary bed), chronic heart failure, primary or secondary pulmonary vascular disease, and restrictive lung disease (e.g., interstitial lung disease [ILD] with reduced capillary bed). Overall, high V/Q mismatch usually has only a minor effect on arterial blood gases (more on CO_2_ than on O_2_ due to impaired release of CO_2_ to the alveoli), resulting in increased P(a-ET)CO_2_, because other lung areas are overventilated in a compensatory manner.

The two extreme variants (V/Q = 0 [complete ventilation disorder; e.g., atelectasis, pneumonia] and V/Q = ∞ [complete perfusion disorder, e.g., acute pulmonary embolism]) do not play a practical role in CPET, because the clinical situation usually precludes exercise testing. **Note:** V/Q mismatch primarily affects PaO_2_ because changes in PaCO_2_ are usually well compensated by hyperventilation in patients with maintained breathing reserve.

### Performing CPET

CPET is typically performed using a cycle ergometer or treadmill [1, 3, 5, 11]. The cycle ergometer is generally safer, is more appropriate for a wide range of patients (e.g., deconditioning, obesity, joint issues), enables more convenient intra-test procedures (monitoring of ECG and blood pressure, blood sampling) and provides an accurate measurement of external work rate. Treadmill ergometry allows subjects to walk or run at measured speeds and grades of incline. The treadmill activates more muscle groups, elicits greater oxygen desaturation and produces higher levels of peak oxygen uptake. In most clinical circumstances, cycle ergometry is the preferable mode of exercise; however, depending on the reason for which CPET is requested, treadmill ergometry may be a suitable alternative [1–3]. Prior to CPET, a precise medical history (pre-existing and concomitant diseases, pacemaker/defibrillator, medications, stimulants, patient activity), clinical examination, basic cardiopulmonary diagnostics (chest X-ray, ECG, blood pressure, body temperature, lung function, TLCO) and laboratory results (e.g., blood count, glucose, creatinine, thyroid-stimulating hormone, blood gas analysis [BGA]) should be available. This facilitates subsequent interpretation and individual risk assessment. Although CPET is considered a safe examination, cardiac emergencies, hypoxaemia and vasovagal/orthostatic syncope can occur. Therefore, qualified staff must be regularly trained for emergency management. A trained physician should be present during testing, at least when at-risk patients are being assessed [23].

CPET is usually performed as symptom-limited cycle ergometry in a sitting position. A continuously incrementing ramp protocol (increase of work rate, e.g., every 2–15 s) or minute-by-minute increments in 5–30 W/min steps to symptom-limited maximum of exercise is used as standard. This offers the advantage of a short protocol with low initial work rate and a brief duration of high-intensity cardiopulmonary exercise.

According to current recommendations [1, 6, 10, 11, 24], the CPET procedure is divided into four parts:

1. Resting phase (2–3 min): adaptation of respiration to the mask or mouthpiece including measurements of capillary BGA, ECG and blood pressure.
2. Unloaded phase (“active baseline”; 2–3 min): unloaded cycling with no added resistance (internal work rate depending on equipment: 0–15 watts), cadence 55–70 revolutions per min (rpm). ${\dot{\text{V}}\text{O}}_{2}$ normally doubles during this warm-up phase.
3. Incremental exercise phase (10 ± 2 min): cadence 55–70 rpm.
4. Recovery phase (cool down period; 3–5 min): unloaded pedalling.

**Note:** unloaded cycling (warm-up) before exercise represents the real cardiopulmonary and metabolic baseline, and should be viewed as an obligatory part of CPET for all patients. Adherence to these guideline recommendations will clearly facilitate standardisation and comparability of CPET results.

### Standards of CPET measurement

Standardised examination procedures as outlined in the recent European Respiratory Society statement on standardisation of CPET in chronic lung diseases contribute very significantly to the data quality and the comparability of measurement results [24]. In that context, quality control, specific training and experience of the qualified staff is essential [23].

To obtain conclusive data, patients must be informed about the procedure (including communication by hand signals during the exercise) and encouraged to apply their full effort [25]. On the day of testing, the patient should take his/her usual medications, wear comfortable sportswear/athletic footwear and have eaten their last light meal at least 2–3 h before the investigation. In addition, the patient should be clinically stable, free of infection and avoid smoking or vaping, intensive sports, and alcohol for 24 h before the examination.

After ensuring the equipment is calibrated and working correctly, the mask (or mouthpiece) and cycle ergometer is attached to the subject, who is then connected to the monitoring equipment. A representative spirometry provides the foundation for determining the maximum voluntary ventilation. After selecting the appropriate incremental ramp protocol, the patient should pedal with a constant cadence (approximately 55–70 rpm). Stopping rules are consistent with those of exercise ECG. At the end of the exercise test, assessment of dyspnoea and leg effort is recorded using a modified Borg CR 10^®^ scale, and the cause(s) of termination are documented.

**Fig. 2:**
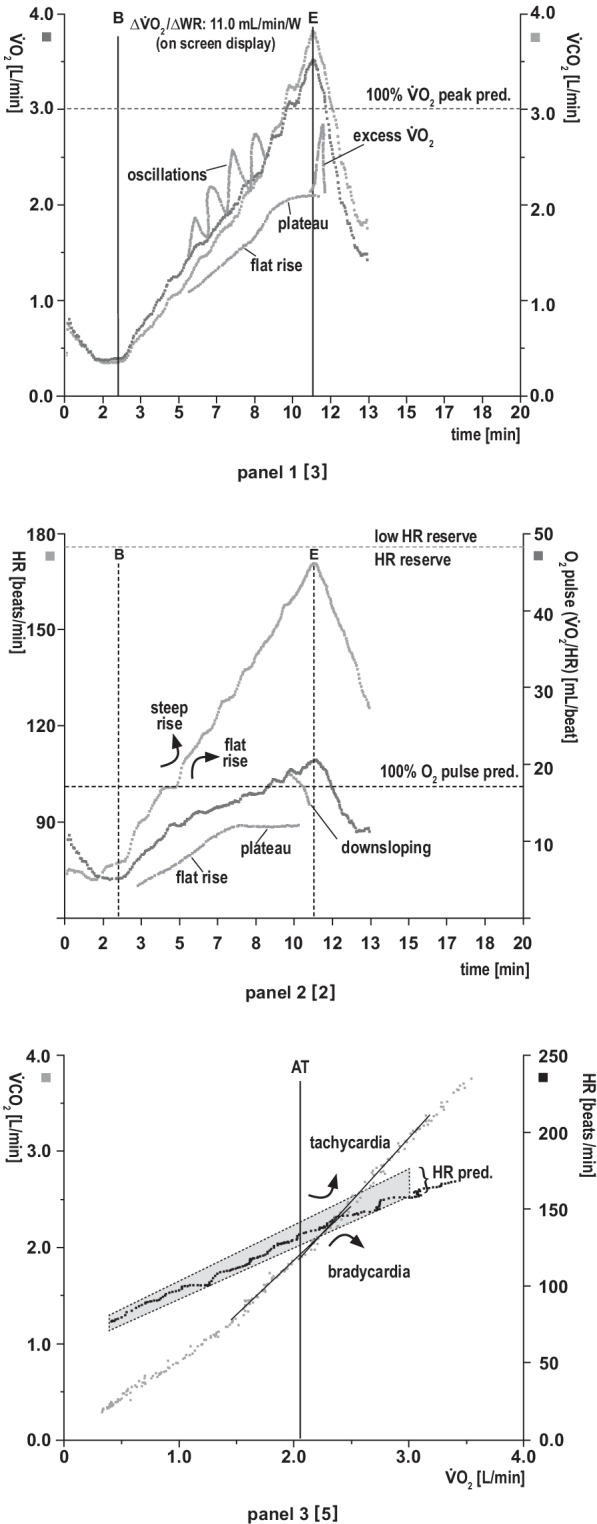
Cardiovascular panels. **Panel 1:** **O**_**2**_ **uptake (VO**_**2**_**) and CO**_**2**_ **output (VCO**_**2**_**) vs. time plus relationship of peak VO**_**2**_ **and work rate (WR)**. B, beginning and E, end of exercise. Peak ${\dot{\text{V}}\text{O}}_{2}$ indicates peak exercise capacity and oxygen uptake at the end of an incremental exercise test. Validity is dependent on patient effort. It is an index of long-term survival. Increase ΔV̇O_2_/ΔWR: provides information about the contribution of aerobic metabolism to exercise (aerobic capacity). A low ratio indicates impaired O_2_ delivery and high anaerobic metabolism during exercise (e.g., peripheral artery, cardiovascular, pulmonary vascular and/or lung disease). Panel [3] refers to the original 9-panel display [26]. **Analysis (target values and response kinetics):** Peak ${\dot{\text{V}}\text{O}}_{2}$ within normal limits or reduced (indicates impaired O_2_ transport and/or utilisation)? Early flattening, reduction or plateau of peak V̇O_2_? Overshoot in ${\dot{\text{V}}\text{O}}_{2}$ following termination of exercise (short-term increase in stroke volume [SV] with reduced afterload; e.g., cardiovascular disease)? Post-exercise ${\dot{\text{V}}\text{O}}_{2}$ recovery to baseline delayed (indicates high O_2_ deficit during exercise)? Δ${\dot{\text{V}}\text{O}}_{2}$-peak/ΔWR during exercise: normal, increased (e.g., obesity) or flattening/downsloping? Oscillatory patterns at rest/moderate exercise (indicates left chronic heart failure [CHF] with poor prognosis)? **Panel 2: Relationship of heart rate and oxygen pulse vs. time**. O_2_ pulse (${\dot{\text{V}}\text{O}}_{2}$/HR) indicates the amount of oxygen extracted by the tissues per heartbeat. This provides information about SV and cardiac output (SV × C(a-$\overline{\upsilon }$) O_2_) during exercise. Heart rate (HR) is the factor that normally limits exercise capacity in healthy subjects. **Analysis (target values and response kinetics):** O_2_ pulse at peak exercise: normal or reduced (impaired transport of O_2_ and/or O_2_ utilisation, e.g., cardiovascular disease, anaemia, peripheral arterial disease [PAD], myopathy) or elevated (chronotropic incompetence, e.g. beta-blocker therapy, heart failure, atrial flutter, tachycardia)? Plateau formation of O_2_ pulse (below predicted value)? Linear or flat increase of O_2_ pulse during early, middle or late exercise? Post-exercise O_2_ pulse recovery to baseline delayed (suggests large O_2_ deficit during exercise)? Increase in HR vs. ${\dot{\text{V}}\text{O}}_{2}$ normal, steep or low (suggests chronotropic incompetence)? HR: elevated at rest? Alternating course of HR during exercise (indicates arrhythmia)? HR reserve (maximal HR predicted—peak HR at peak ${\dot{\text{V}}\text{O}}_{2}$): normal, high or low? **Panel 3: Relationships of CO**_**2**_ **output (**${\dot{{\mathbf{V}}}\mathbf{CO}}_{2}$**) (y-axis) and O**_**2**_ **uptake (**${\dot{{\mathbf{V}}}\mathbf{O}}_{2}$ **(x-axis) and the relationship between HR and** ${\dot{{\mathbf{V}}}\mathbf{O}}_{2}$. First reference to determine AT (see main text). AT corresponds to the curve point at which, due to CO_2_-related hyperventilation, ${\dot{\text{V}}\text{CO}}_{2}$ begins to continuously rise more steeply than the ${\dot{\text{V}}\text{O}}_{2}$ (V-slope method). The V-slope is less responsive to breathing irregularities than P_ET_O_2_ and ${\dot{\text{V}}\text{E}}/{\dot{\text{V}}\text{O}}_{2}$. HR: more detailed information on HR behaviour (incl. target value range) See also HR at Panel 2. **Analysis (target values and response kinetics)**: AT in target range or reduced (indicates impaired O_2_ delivery)? Cross-check with panels 4, 7 (3-panel view). Linear increase in HR relative to ${\dot{\text{V}}\text{O}}_{2}$? HR reserve: normal, low or increased?

**Fig. 3:**
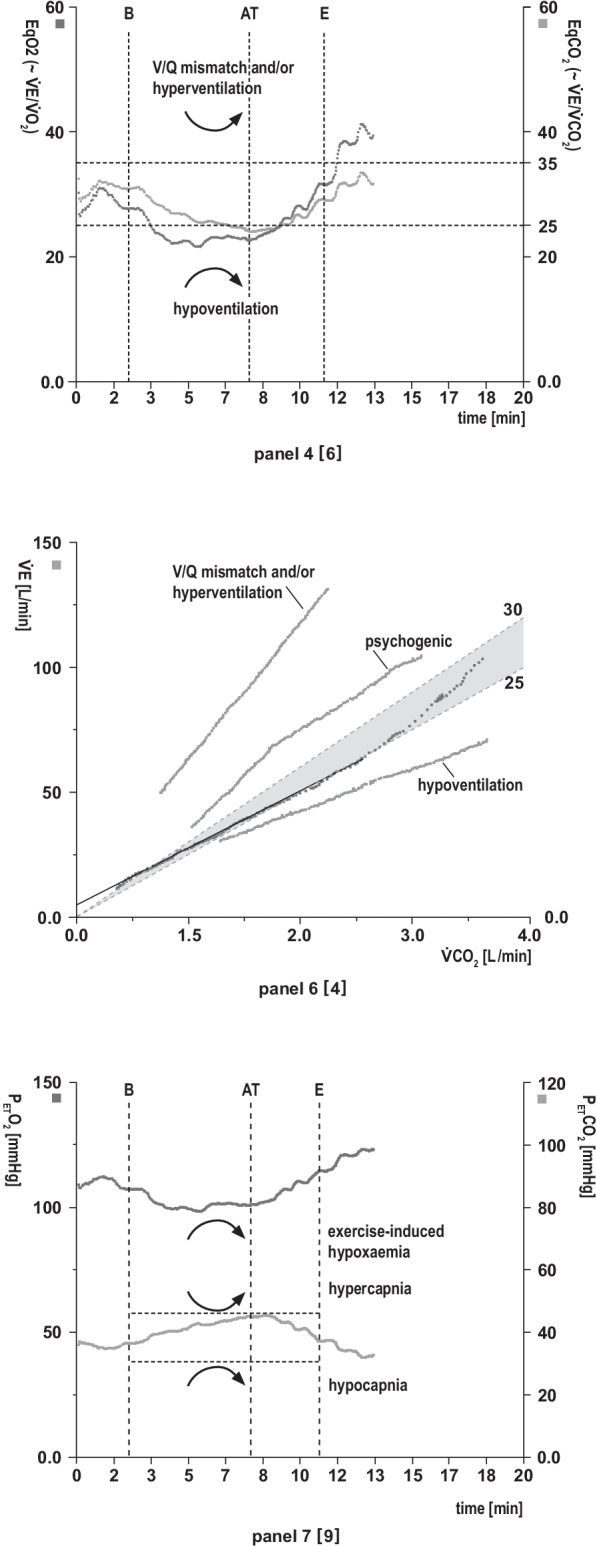
Pulmonary gas exchange panels **Panel 4:** **The relationships of minute ventilation (**${\dot{{\mathbf{V}}}\mathbf{E}}$**) vs. O**_**2**_** uptake (**${\dot{{\mathbf{V}}}\mathbf{O}}_{2}$**) and vs. CO**_**2**_ **(**${\dot{{\mathbf{V}}}\mathbf{CO}}_{2}$**) output (ventilatory equivalents) as a function of time**. The ventilatory equivalents EqO_2_ ≈ ${\dot{\text{V}}\text{E}}/{\dot{\text{V}}\text{O}}_{2}$ and EqCO_2_ ≈ ${\dot{\text{V}}\text{E}}/{\dot{\text{V}}\text{CO}}_{2}$ indicate how many litres must be ventilated to take up 1 L of O_2_ or exhale 1 L of CO_2_ (gas exchange efficiency). The same information is found in panel 6 in a linear presentation. The lower the equivalent values, the more effective the gas exchange or work of breathing, and vice versa. Excess ${\dot{\text{V}}\text{E}}$ vs. ${\dot{\text{V}}\text{O}}_{2}$ and ${\dot{\text{V}}\text{CO}}_{2}$ occurs due to augmented ventilatory drive (nonspecific hyperventilation), metabolic acidosis (compensatory hyperventilation) and/or V/Q mismatch (true ventilatory inefficiency). Additional possibility of determining the AT. AT corresponds to the lowest point (nadir) of EqO_2_ directly before EqO_2_ continuously increases (provided EqCO_2_ does not increase simultaneously). **Analysis (target values and response kinetics):** Physiological decrease in EqO_2_ and EqCO_2_ from rest to AT? Significantly elevated EqO_2_ and EqCO_2_ values at rest or during exercise? Significantly decreased EqO_2_ and EqCO_2_ values (indicates alveolar hypoventilation)? AT within predicted values or reduced? Cross-validate with panels 3, 7 (3-panel view). **Panel 6: The relationship of ventilation (**${\dot{{\mathbf{V}}}\mathbf{E}}$**) and CO**_**2**_** production (**${\dot{{\mathbf{V}}}\mathbf{CO}}_{2}$**):** ${\dot{\mathbf{V}}\mathbf{E}}/{\dot{\mathbf{V}}\mathbf{CO}}_{2}$ **slope**. The ${\dot{\text{V}}\text{E}}/{\dot{\text{V}}\text{CO}}_{2}$ slope is a measure of ventilatory (gas exchange) efficiency at submaximal exercise. The same information can be found in panel 4 (EqCO_2_ ≈ ${\dot{\text{V}}\text{E}}/{\dot{\text{V}}\text{CO}}_{2}$) in a nonlinear presentation, but values are not identical [21]. The ${\dot{\text{V}}\text{E}}/{\dot{\text{V}}\text{CO}}_{2}$ slope is a prognostic indicator in CHF. **Analysis (target values and response kinetics):** ${\dot{\text{V}}\text{E}}/{\dot{\text{V}}\text{CO}}_{2}$ slope within normal range (preserved V/Q matching)? Steep increase in the ${\dot{\text{V}}\text{E}}/{\dot{\text{V}}\text{CO}}_{2}$ slope indicative of significant V/Q mismatching (${\dot{\text{V}}\text{E}}/{\dot{\text{V}}\text{CO}}_{2}$ slope ≥ 39 [27]) and/or nonspecific/compensatory hyperventilation (which is usually paralleled by ↑PETO_2_ and ↓PETCO_2_)? Initial sharp increase in the ${\dot{\text{V}}\text{E}}/{\dot{\text{V}}\text{CO}}_{2}$ slope that levels off with increasing work rate (suggestive of psychogenic hyperventilation)? Decrease in the ${\dot{\text{V}}\text{E}}/{\dot{\text{V}}\text{CO}}_{2}$ slope indicates alveolar hypoventilation. **Panel 7: End-tidal partial pressures of O**_**2**_ **(P**_**ET**_**O**_**2**_**) and CO**_**2**_ **(P**_**ET**_**CO**_**2**_**) vs. time**. Indirect measure of pulmonary gas exchange and V/Q mismatch. The more pronounced the ventilation, the lower the P_ET_CO_2_ and the higher the P_ET_O_2_, and vice versa in normal lungs. (Note: P_ET_CO_2_ ≠ PaCO_2_. P_ET_CO_2_ > PaCO_2_ during exercise (approx. 4 mmHg); at rest: P_ET_CO_2_ < PaCO_2_ (approx. 2 mm Hg). Additional possibility of determining the AT. AT corresponds to the lowest point (nadir) of P_ET_O_2_ directly before P_ET_O_2_ continuously increases (provided P_ET_CO_2_ remains constant). **Analysis (target values and response kinetics)**: Physiological course of P_ET_O_2_ and P_ET_CO_2_ at rest and during exercise? Cross-check with BGA. AT within predicted values or reduced? Cross-check with panels 3, 4 (3-panel view). Decrease in P_ET_O_2_ (indicates exercise-induced hypoxaemia) or abrupt increase at start of exercise (may indicate R-L-shunt or nonspecific hyperventilation)? Significant drop in P_ET_CO_2_ during exercise (suggests V/Q mismatch and/or hyperventilation)? Significant increase in P_ET_CO_2_ during exercise (indicates alveolar hypoventilation, e.g., severe COPD, obesity hypoventilation syndrome, neuromuscular disease)? **Note:** the determination of P(A-a)O_2_ or P(a-ET)CO_2_ more sensitively and reliably identifies and quantifies low or high V/Q regions (and/or a R-L-shunt) than end-tidal partial pressures

**Fig. 4:**
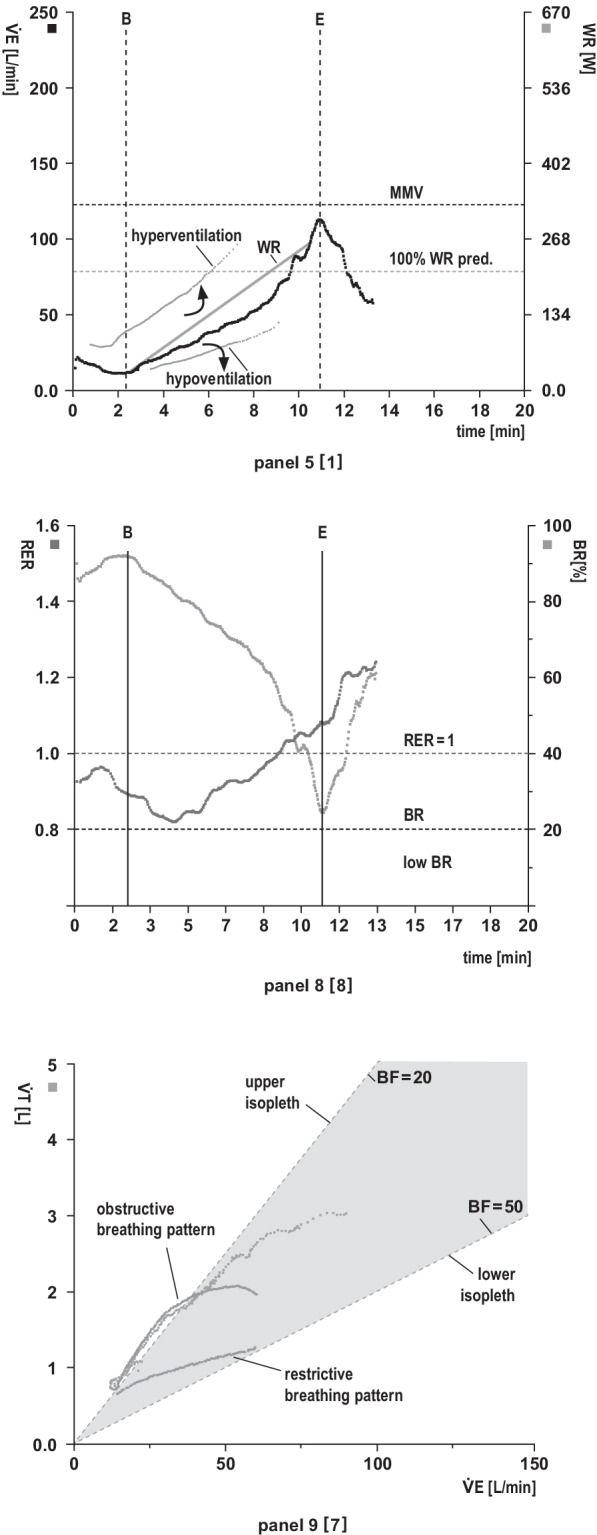
Ventilatory response panels **Panel 5: Relationship between minute ventilation (**${\dot{{\mathbf{V}}}\mathbf{E}}$**) and work rate (WR) vs. time (x-axis)**. The maximum voluntary ventilation (MVV) is calculated indirectly as forced expiratory volume in 1 s (FEV_1_) × 40 or can be determined by direct measurement of MVV (preferred option in restrictive lung disease). Exercise is usually not limited by breathing. **Analysis (target values and response kinetics):** Is ${\dot{\text{V}}\text{E}}$ adequate relative to work rate (see main text: validity check, 9-point rule)? Is ${\dot{\text{V}}\text{E}}$ vs. work rate sharply increased at the start of exercise (suggestive of R-L shunting) or decreased (e.g., mask or mouthpiece leakage)? Impaired ability to increase ${\dot{\text{V}}\text{E}}$ in response to enhanced CO_2_ production and/or acidaemia (e.g., severe lung disease, obesity)? **Panel 8: Respiratory exchange rate (RER) and breathing reserve (BR).** RER describes the ratio of CO_2_ output to O_2_ uptake (${\dot{\text{V}}\text{CO}}_{2}$/${\dot{\text{V}}\text{O}}_{2}$) as a function of time and reflects patient effort (RER at least ≥ 1). RER depends on the rate of lactate increase during progressive exercise. BR indicates the actual percentage of the maximum ventilatory capacity (MVV-V̇E). Validity depends on adequate spirometry. **Analysis (target values and response kinetics):** RER values at rest: normal, high or low? RER > 1 at rest (indicative of hyperventilation). RER ≥ 1 achieved in early exercise (work rate already above lactate threshold) or in late exercise? An abrupt, persistent RER increase during early exercise suggests exercise-induced R-L shunt. RER < 1 during exercise (e.g., poor effort, severe lung disease [${\dot{\text{V}}\text{E}}$ cannot be adequately increased], myopathy, PAD, hyperventilation prior to testing)? Delayed decrease in RER in early recovery (indicates delayed CO_2_ elimination, e.g., severe COPD) or rapid RER decrease in early recovery (indicates delayed recovery of ${\dot{\text{V}}\text{O}}_{2}$ vs. ${\dot{\text{V}}\text{CO}}_{2}$ due to a high O_2_ deficit during exercise)? BR normal or low? A low BR indicates reduced ventilatory capacity due to impaired lung mechanics and increased ventilatory demands during exercise. ** Panel 9: Breathing pattern. Relationships of tidal volume (V**_**T**_**) (y-axis), minute ventilation (**${\dot{{\mathbf{V}}}\mathbf{E}}$**) (x-axis) and breathing frequency (BF)**. BF is indirectly presented in the form of isopleths (= line with the same numerical values. Upper isopleth: low BF [= 20 breaths/min]. Lower isopleth: high BF [= 50 breaths/min]). Physiologically, V̇E increases until V_T_ is fully utilised (≈ 60% of VC), thereafter V̇E increases with a rise in BF. **Analysis (target values and response kinetics): Normal breathing pattern?** The values in the area of the upper isopleths indicate a high V_T_ and a low BF. **Obstructive breathing pattern?** The increase in ventilation during exercise is limited because V_T_ is already fully utilised and eventually falls off. BF cannot be adequately increased due to the prolonged expiration time. This results in slow, deep breathing. **Restrictive breathing pattern?** The increase in ventilation during exercise is limited because V_T_ cannot be sufficiently increased due to the reduced lung volume (VT/IC ratio ↑ [> 0.8]). Hence, ventilation can only be increased by elevated BF. V_T_ runs low and flat in the direction of the lower isopleth. This results in rapid, shallow breathing.

During CPET, the O_2_ and CO_2_ concentrations of exhaled air and minute ventilation (${\dot{\text{V}}\text{E}}$) (tidal volume × breathing frequency) are continuously measured via the face-mask (or mouthpiece) with connected gas and flow (or volume) sensors. From these measurements and exercise test monitoring (heart rate and work rate), several key variables can be derived (Figs. 1, 2, 3 and 4).

The data are collected for each individual breath (single breath analysis), averaged over 8–10 breaths (rolling averages) or averaged over a fixed period of 10–30 s and graphically displayed as a tabular summary and a 9-panel graphical array. The graphical display, parameter selection and scaling are preconfigured and can be adapted to investigator requirements with the support of the manufacturer. A number of formatting conventions regarding the 9-panel plots have been proposed to improve the clarity, reproducibility, interpretation and comparability of CPET results [28]. For reference values [29], we prefer to use the equations from the SHIP cohort [30] or, alternatively, the similar values from Hansen/Wasserman [31].

In addition, capillary BGA [time points: at rest, sub-maximum exercise (in the range of AT), peak exercise, end of recovery (e.g., 2 min post-exercise)] from a hyperaemic earlobe by trained staff is recommended to quantify the amount of potential V/Q mismatch [determination of P(A-a) O_2_ and of P(a-ET) CO_2_]. This is in line with current practice and recent recommendations de-emphasising the need for arterial BGA samples for CPET in non-hypoxaemic patients [1, 32]. However, differences between capillary and arterial PO_2_ (usually in the range of 5–10 mm Hg) in patients with oxygen saturation (SpO_2_) > 90% should be kept in mind.

### Selection of the individual work rate

Incremental exercise should ideally last for 10 ± 2 min, or for at least 5 min in severely restricted patients. The selection of a work rate increment that is too rapid (ramp too steep) should be avoided because this is often associated with marked hyperventilation, an inability to determine AT and premature termination of exercise due to lactate acidosis. Thus, selecting a work rate increase of 5 watts/min in significantly impaired patients might be worth considering.

There are several options when selecting the total workload (watts) and rate of work rate increase (watts/min). Basically, a simple orientation on exercise capacity in everyday life has proven to be useful and pragmatic: the staircase question (e.g., how many floors can you walk up quickly without stopping? [11, 33]. Responses can inform CPET ramp modifications as follows:

- One floor is equivalent to approximately 50 watts (≈ 5 W/min for a 10-min test) and corresponds to easy hiking, playing golf, etc. For patients with severe cardiac and/or lung disease, 50 watts may represent their maximum exercise capacity, therefore, assign a work rate increment of 5 W/min to achieve a 10-min test.
- Two floors is equivalent to approximately 100 watts, corresponding to “Nordic Walking”, cycling on a flat track, gardening etc. Suggested work rate increment is 10 W/min.
- Three floors is equivalent to approximately 125–150 watts, corresponding to swimming, mountain hiking, etc. Suggested work rate increment is 15 W/min.
- Four floors is equivalent to approximately 200 watts (similar to running at ≥ 10 km/h). Suggested work rate increment is 20 W/min.

Alternatively, other work rate increment estimates have been proposed in the literature [1, 24].

### Validity check

A validity check is mandatory to detect and correct equipment malfunctions before and during CPET (e.g., mask leakage, defect or drift of the gas analysers). A simple validity check can be limited to the following considerations [11]:

- Adequate minute ventilation? Implausible if the increase of ${\dot{\text{V}}\text{E}}$ does not follow an increase in work rate (mask leakage, anxiety, poor effort?). For rapid estimation of an adequate ${\dot{\text{V}}\text{E}}$ relative to work rate we suggest using the 9-point rule described by Rühle [11]: each 25 W increase in work rate requires 9 L of ${\dot{\text{V}}\text{E}}$ plus 9 L of ${\dot{\text{V}}\text{E}}$ at rest. Example: a total work rate of 100 W (4 × 25) requires a ${\dot{\text{V}}\text{E}}$ of 4 × 9 L + 9 L (at rest) = 45 L/min.
- Adequate ${\dot{\text{V}}\text{O}}_{2}$ increase for a given work rate (${\dot{\text{V}}\text{O}}_{2}$ increase/WR)? Implausible if the increase during early exercise (first 1–2 min) is too low (e.g., mask leakage). The actual value can be read online on the screen. Rule of thumb: ${\dot{\text{V}}\text{O}}_{2}$ increase/WR ≥ 10 mL/min/watt. A value of 5–6 mL/min/kg is suitable as a plausibility check at rest for ${\dot{\text{V}}\text{O}}_{2}$ (rule of thumb).
- Adequate respiratory exchange rate (RER)? Implausible if RER at rest is < 0.7 or RER at early exercise is > 1 (volitional or anticipatory hyperventilation, gas analyser malfunction, clogged sample tube, mask leakage).

Possible solutions: follow the manufacturer’s instructions for the warm-up period of the measuring equipment, repeat calibration, check gas cartridge, exchange (mask, measuring sensors, sample tube). Relevant artefacts also occur if the marked termination of the incremental exercise period in the software does not match the actual end of active exercise on the ergometer. Another common error is a faulty capillary BGA sample (erroneous blood sample extraction or analysis, missing or incorrect marking or incorrect order of the BGA entries in the software program).

### Adequate patient effort

Parameters such as end-exercise values of RER ≥ 1.05 (ill person) or ≥ 1.1 (healthy people), exceeding the ${\dot{\text{V}}\text{O}}_{2}$ at anaerobic threshold (AT) and coming close to the maximal predicted values of ${\dot{\text{V}}\text{O}}_{2}$ peak, heart rate and ${\dot{\text{V}}\text{E}}$ (and/or ${\dot{\text{V}}\text{E}}$/${\dot{\text{V}}\text{O}}_{2}$ > 30–35) suggest sufficient patient effort. Importantly, CPET should not be stopped when these criteria are met.

### Analysing the 9-panel plot array

The analysis and interpretation of CPET results requires the basic knowledge of exercise physiology along with a structured approach. Regarding exercise capacity/performance, the maximum achieved exercise performance (${\dot{\text{V}}\text{O}}_{2}$ peak = highest oxygen uptake upon discontinuation of exercise) is more relevant than the maximum attainable exercise capacity (${\dot{\text{V}}\text{O}}_{2}$ max), which is the domain of sports medicine.

Key variables and their interrelationships are systematically summarised in the 9-panel display of Wasserman et al. [26]. The plot enables a reliable, structured interpretation and a feasible distribution of test results. In 2012, Wasserman et al. rearranged the original 9-panel display, with identical content, for didactic reasons [34]. However, the updated display has not yet become generally established, so we will refer to both versions.

The primary objective of the interpretation is to determine whether and to what extent there is impaired exercise capacity and what cause(s) of cardiovascular, pulmonary vascular or pulmonary origin may be primary.

It has proven useful to analyse the 9-panel display in a systematic order across the entire period of testing (at rest, exercise and recovery) [1, 3, 11, 19, 20]. Information on the cardiovascular response and oxygen transport is reflected in panels 1 → 2 → 3 (original version: panels 3 → 2 → 5).

Information on pulmonary gas exchange and V/Q mismatch can be found in panels 4 → 6 → 7 (original version: panels 6 → 4 → 9). A possible limitation of ventilatory capacity is shown in panels 5 → 8 → 9 (original version: panels 1 → 8 → 7).

In view of the large number of CPET variables (approximately 150), the reduction to a few clinically meaningful key variables is truly remarkable and also enables non-specialists to perform a structured analysis and interpretation using the 9-panel display.

In Figs. 2, 3 and 4, we describe the individual panels in the above-mentioned sequential order using the example of a normal finding in a healthy 44-year-old man who performed cycle ergometry with an incremental ramp protocol. The panels thus reflect the physiological changes during exercise as summarised in Fig. 1. The individual panels contain  additional information on the key variables, suggestions for structured analysis and embedded examples of possible abnormal reaction patterns.

### Determination of the anaerobic threshold (AT)

Measurement of the AT allows the objective assessment of aerobic metabolism at submaximal exercise levels. It can be automatically calculated by computer programme but needs to be cross-checked. A low AT indicates impaired cardiovascular transport of oxygen or poor muscular oxygen utilisation. By combining several methods (so-called 3- panel view (panels 3, 4, 7 [original version: panels 5, 6, 9]), AT can be determined in most cases and excludes non-physiologic hyperventilation as the origin of the V-slope inflection point [12]. Valid determination of AT is not always possible, as has been shown for very severe respiratory limitations (COPD, ILD) or significant heart failure when ventilation and/or perfusion can no longer be adequately increased in response to increasing exercise. Other reasons include an excessively steep/mild incremental ramp protocol or performance-reducing factors such as arthrosis, peripheral arterial disease or poor effort. We have not considered the second gas exchange threshold [respiratory compensation point (RCP)] due to its relatively low clinical worth.

Table 1 summarises normal and abnormal values of some central CPET parameters, knowledge of which can be useful for data interpretation. The values are meant for orientation only, because no generally accepted target values have been yet established.

**Table 1:** Suggested target values for key cardiopulmonary exercise testing variables (cycle ergometry) [1, 5–7, 10, 11, 15]

Variable	Target value	Abnormal
Peak ${\dot{\text{V}}\text{O}}_{2}$ (exercise capacity)	≥ 85% based on ${\dot{\text{V}}\text{O}}_{2}$ pred. or > 20 mL O_2_/min/kg	< 85%/ < 70%/ < 50% (mild/moderate/severe)
${\dot{\text{V}}\text{O}}_{2}$/WR (aerobic capacity)	≥ 9–10 mL/min/watt^1^	≤ 8 mL/min/watt
${\dot{\text{V}}\text{O}}_{2}$ at AT	≥ 40–80% pred. ${\dot{\text{V}}\text{O}}$ (usually 50–65% of peak ${\dot{\text{V}}\text{O}}_{2}$)	< 40%/ < 30%/ < 25% (mild/moderate/severe)
Blood pressure	Increase by 10 mmHg per 30 watts	Decrease, inadequate increase
O_2_ pulse (${\dot{\text{V}}\text{O}}_{2}$/HR)^2^	≥ 80%	< 70% pred. during peak exercise
Heart rate reserve (HRR)	≥ 85% pred. (< 15 bpm)	< 85% predicted (but wide range)
Breathing reserve (BR)	≥ 15–20% (or ≥ 11–15 L/min)	< 15–20% (or < 11–15 L/min)
Breathing frequency (BF)	≤ 50/min	≥ 60/min
EqCO_2_ at AT	25–30 at AT, ≤ 40 after AT	≥ 35 at AT, > 40 after AT;
EqO_2_ at AT	20–30 at AT, ≤ 40 after AT	≥ 35 at AT, > 40 after AT
${\dot{\text{V}}\text{E}}/{\dot{\text{V}}\text{CO}}_{2}$ slope	25–30 (slightly lower than EqCO_2_ at AT)	≥ 35 or < 20
RER	≥ 1.05 (ill) or ≥ 1.1 (healthy); > 1.1–1.5 in recovery phase; at rest: > 0.7, < 1.0	< 1 (peak exercise)
PETCO_2_ (≈ PACO_2_ ≈ PaCO_2_)	> 35 mmHg (at rest); > 40 mmHg (during exercise)	< 33 mmHg (at rest), < 3 mmHg increase or > 50 mmHg (peak exercise)
PETO_2_ (≈ PAO_2_)	≥ 90 mmHg (at rest), 20–30 mmHg increase during exercise	Lack of increase or decrease during exercise
P(A-a) O_2_^3^	20 mmHg (at rest); 30 mmHg (during exercise)	> 35 mmHg
P(a-ET) CO_2_^4^	At rest: minimally positive; during exercise: slightly negative	> 5 mmHg

*a* arterial, *A* alveolar, *AT* anaerobic threshold, *bpm* beats/minute, *CO*_*2*_ carbon dioxide, *Eq* ventilatory equivalent, *ET* end-tidal, *HR* heart rate, *O*_*2*_ oxygen, *P* pressure, *pred.* predicted, *RER* respiratory exchange rate, ${\dot{\text{V}}\text{E}}/{\dot{\text{V}}\text{CO}}_{2}$ ventilatory equivalent for carbon dioxide, ${\dot{\text{V}}\text{O}}_{2}$ oxygen uptake, *WR* work rate

^1^Peak ${\dot{\text{V}}\text{O}}_{2}$ in obesity should be expressed as L/min or referenced to weight predicted

^2^O_2_ pulse (V̇O_2_/HR) peak exercise values vary widely by the same factors that affect normal peak ${\dot{\text{V}}\text{O}}_{2}$ and HR (e.g., age, body size, gender, Hb concentration, work rate, fitness level)

^3^P(A-a)O_2_ indicates efficacy of O_2_ uptake. PAO_2_ is calculated using the alveolar air formula (requires PaCO_2_ from blood gas analysis [BGA]), PaO_2_ is also determined using BGA

^4^P(a-ET)CO_2_ indicates efficacy of CO_2_ output to the alveoli: PACO_2_ is measured as PETCO_2_; PaCO_2_ is determined by BGA. P(a-ET)CO_2_ is slightly positive at rest due to V/Q inhomogeneities (poorly perfused upper lung areas with impaired CO_2_ production, PaCO_2_ > PACO_2_), and negative due to hyperventilation during exercise (PaCO_2_ < PACO_2_ [difference approximately 4 mmHg])

### Interpretation of CPET results

Exercise capacity in healthy subjects is normally limited by the heart or the musculoskeletal system. In patients complaining of exercise intolerance, a CPET can often reveal the primary source of exercise limitation that, together with the results from clinical history (including neurological disorders [autonomic dysfunction]) and resting functional diagnostics can narrow down a broad differential diagnosis and diagnostic options.

Table 2 shows an example of a CPET interpretation worksheet that can guide structured interpretation of the data and determine the primary pattern of exercise limitation (e.g., cardiocirculatory, pulmonary vascular, pulmonary, deconditioning).

**Table 2:** Identification of abnormal reaction pattern(s)

Individual findings	Patient	Cardiovascular	Pulmonary vascular	Pulmonary	Lack of fitness
Reduced peak ${\dot{\text{V}}\text{O}}_{2}$		X	X	X	X
Low ${\dot{\text{V}}\text{O}}_{2}$ at AT		X	X	X	(X)
Steep HR increase relative to ${\dot{\text{V}}\text{O}}_{2}$ and shallow rise in O_2_ pulse, respectively		X	X		X
Low ${\dot{\text{V}}\text{O}}_{2}$/WR slope during incremental exercise		X	X		
Elevated ${\dot{\text{V}}\text{E}}/{\dot{\text{V}}\text{CO}}_{2}$ slope or elevated EqCO_2_ at AT		*	X	X	
Normal breathing reserve		X	X		X
ECG changes, inadequate BP behaviour		X			
Low peak HR				X	X
Low PETCO_2_ or PaCO_2_ at rest and/or decrease during exercise		*	X		
SpO_2_ or PaO_2_ decrease during exercise			X	(X)	
Low breathing reserve				X	
Abnormal breathing pattern**				X	

Distinction between cardiovascular, pulmonary vascular and pulmonary reaction patterns or deconditioning as the primary cause(s) of exercise limitation (modified according to [1, 3, 7, 10, 20]). In the table, the findings that apply individually can be check-marked in the patient column. The identified pathophysiology can be used to establish the likely primary cause of the individual exercise limitation. Overlaps can occur between the categories (e.g., in chronic lung diseases with secondary effects on pulmonary vascular and myocardial function or in comorbid disease states). It should be noted that the severity of the underlying disorder has a major influence on the reaction patterns (e.g., normal peak ${\dot{\text{V}}\text{O}}_{2}$ in mild to moderate asthma). Exercise intolerance by claudications, pain or muscle fatigue etc. support the clinical suspicion of peripheral artery disease (PAD) or myopathies

*a* arterial, *AT* anaerobic threshold, *BP* blood pressure, *CO*_*2*_ carbon dioxide, *ECG* electrocardiogram, *Eq* ventilatory equivalent, *ET* end-tidal, *HR* heart rate, *O*_*2*_ oxygen, *P* pressure, *SpO*_*2*_ oxygen saturation, ${\dot{\text{V}}\text{E}}/{\dot{\text{V}}\text{CO}}_{2}$ ventilatory equivalent for carbon dioxide, ${\dot{\text{V}}\text{O}}_{2}$ oxygen uptake, *WR* work rate

*Moderate to severe left ventricular failure. Patients with myocardial ischaemia without chronic heart failure, mild left ventricular failure, PAD, anaemia, deconditioning and poor effort alone demonstrate normal V/Q ratios and normal values for P_ET_CO_2_, PaCO_2_

**Restrictive or obstructive breathing pattern, not including exercise oscillatory ventilation

The worksheet approach is certainly not the only primer for a physiologically-based interpretation of exercise intolerance (e.g., see structured flowchart approach [1]), but offers the advantage that in most cases it can help clarify which is/are the primary pattern(s) of exercise limitation [20]. As illustrated in Table 2, a statement can be made from the sum of individual findings for each category. In addition, overlaps can occur between the categories, but usually a clear distinction can be made as to which of the categories—cardiac, pulmonary vascular or pulmonary—is dominant and whether there is evidence of secondary effects (e.g., pulmonary hypertension) or coexistent disease that may affect outcomes. Consequently, this analysis might lead to unexpected previously unknown causes of exercise intolerance that cannot be determined without CPET. Finally, CPET may also be useful in confirming normal findings that make significant disease unlikely.

It is also important to note that an individual with normal peak ${\dot{\text{V}}\text{O}}_{2}$ may still have exercise limitation caused by deconditioning, obesity, anxiety or early/mild cardiopulmonary disease. Other confounding factors such as anaemia, thyroid function or acid–base disorders should be investigated before the examination.

### Exercise ECG and blood pressure

During the entire examination, attention must be paid to abnormalities in blood pressure and ECG (ST changes, arrhythmias, ectopic beats and block patterns).

### CPET reporting

Suggestions for a CPET report have been described in the literature [1, 5–7, 24]. The possibilities of computer-assisted report generation are often underutilised, but this would be likely to improve the necessary timeframe, the interpretation and quality of the test report.

### Intrabreath curves (IC manoeuvre)

The non-standardised registration of intrabreath or inspiratory capacity (IC) manoeuvres during exercise (times of measurement: at rest, at moderate exercise (before AT), at peak exercise) can provide additional information about respiratory mechanics (dynamic hyperinflation, expiratory flow limitation) in condition-specific cases (e.g., obstructive lung disease, pulmonary vascular disorders) [2, 7, 11, 35]. The IC manoeuvre should not be confused with that of exercise-induced bronchoconstriction [36].

## Conclusion

CPET provides an objective and reproducible opportunity to identify why an individual is complaining of exertional dyspnoea and to quantify the limitation of exercise capacity. It can help not only to differentiate between pulmonary, pulmonary vascular and cardiovascular disease but also to unmask the underlying and often complex mechanisms. Accordingly, CPET should be performed before the patient undergoes extensive diagnostic workup that searches in a state of rest for an abnormality that takes place during exercise. CPET probably covers a broader range of potential differential diagnoses than any other test in medicine and is also likely to be cost effective because it directs diagnosis and facilitates treatment decisions [1]. Moreover, many patients regard CPET as being a very useful part of their clinical examination [24]. This all suggests that CPET should be used much more frequently, particularly since the expenditure of time, e.g., compared to exercise ECG, is low in routine use. In addition, the diagnostic value of CPET significantly exceeds that of non-discriminating tests of exercise performance (exercise ECG, 6-min walking test, etc. that provide no information about exercise tolerance), because prognostically important key variables can be determined with the simultaneous measurement of ventilatory gas exchange, even at submaximal exercise levels. However, this global cardiopulmonary reference test is increasingly at risk of disappearing from outpatient specialist medical care for a variety of reasons, such as cost, lack of expertise or reimbursement [15]. This inconsistency is partly explained by the fact that CPET statements may be considered complicated and often fail to provide practical, easy-to-follow guidance [6]. CPET can be seen as a complex test (based on the unique wealth of information it provides) but not necessarily a difficult tool that can be performed well by non-specialists. However, lack of a compact and readily accessible introduction for those interested in learning how to analyze and interpret CPET findings might limit wider use of this powerful reference method. Accordingly, CPET should be promoted in the clinical setting and training should be a mandatory component of respiratory specialist medical training. In this regard, the exemplified CPET standard operating procedure of the German Centre for Cardiovascular Research recommends the initial guided application of 5 CPETs and the subsequent independent performance and interpretation of at least 20 CPETs under supervision [37]. Although this introduction is not intended to be comprehensive, we have attempted to provide a practical guide for those involved in the performance and interpretation of CPET, and to encourage the use of this specialist reference examination much more frequently in indicated cases.

## Supplementary Information

**Additional file 1.** Exercise physiology.

## Data Availability

Not applicable.

## Abbreviations

AT: Anaerobic threshold
B: Beginning of exercise
BF: Breathing frequency
BGA: Blood gas analysis
BR: Breathing reserve
CAD: Coronary artery disease
CaO_2_: Arterial oxygen concentration (arterial content O_2_ = Hb × 1.39 (Hüfner number) × SpO_2_% [g O_2_ per 100 mL blood]
${\text{C}}\left( {{\text{a}} - \bar{\upsilon }} \right){\text{O}}_{2}$: Difference between arterial and mixed venous O_2_ content
CHF: Chronic heart failure
COPD: Chronic obstructive pulmonary disease
COVID-19: Coronavirus disease 2019
CPET: Cardiopulmonary exercise testing
E: End of exercise
ECG: Electrocardiogram
EqCO_2_ or EqO_2_: Ventilatory equivalents for carbon dioxide or oxygen
Hb: Haemoglobin
Hg (mm): Mm mercury column
HR: Heart rate (beats/min)
HRR: Heart rate reserve
IC: Inspiratory capacity
ILD: Interstitial lung diseases
L: Liter
MVV: Maximum voluntary ventilation
PAD: Peripheral arterial disease
PACO_2_, PAO_2_: Alveolar partial pressure of carbon dioxide or oxygen
PaCO_2_, PaO_2_: Arterial partial pressure of carbon dioxide or oxygen
${\text{C}}\left( {{\text{a}} - \bar{\upsilon }} \right){\text{O}}_{2}$: Mixed venous partial pressure of carbon dioxide or oxygen
P(A-a)O_2_: Alveolar-arterial partial pressure difference for oxygen
P(a-ET)CO_2_: Arterio-end tidal partial pressure difference for carbon dioxide
P_ET_CO_2_, P_ET_O_2_: End-tidal pressure of carbon dioxide or oxygen at the end of exhalation
RER: Respiratory exchange rate
R-L shunt: Right-left shunt
RCP: Respiratory compensation point
SpO_2_: Peripheral oxygen saturation
SV: Stroke volume
TLCO: Diffusion capacity (transfer factor)
VC: Vital capacity
$${\dot{\text{V}}\text{E}}$$: Minute ventilation
${\dot{\text{V}}\text{CO}}_{2}$: Carbon dioxide production
$${\dot{\text{V}}\text{O}}_{2}$$: Oxygen uptake
${\dot{\text{V}}\text{O}}_{2}$ max: Maximum achievable oxygen uptake
${\dot{\text{V}}\text{O}}_{2}$ peak: Oxygen uptake achieved at end of exercise
${\dot{\text{V}}\text{O}}_{2}$ at AT: Oxygen uptake at the anaerobic threshold
${\dot{\text{V}}\text{O}}_{2}$/HR: Oxygen pulse (mL/min)
${\dot{\text{V}}\text{O}}_{2}$/WR: Aerobic capacity
V_T_: Tidal volume
V/Q: Ventilation/perfusion ratio
WR: Work rate
