# Supplementary material for: Practical guide to cardiopulmonary exercise testing in adults
Source: Respir Res. 2022 Jan 12;23:9. doi: 10.1186/s12931-021-01895-6 (PMC8754079; doi:10.1186/s12931-021-01895-6)
Supplement: Supplementary file 1 — Additional file 1. Exercise physiology. [file 12931_2021_1895_MOESM1_ESM.docx]

**Additional file 1: Exercise physiology**

**Aerobic metabolism**

The increasing energy requirements during exercise are mainly covered by aerobic glycolysis and lipolysis until the anaerobic threshold (AT) is reached.

As work rate increases, oxygen uptake (V̇O_2_) and carbon dioxide production (V̇CO_2_) increase. The V̇O_2_ uptake usually exceeds the V̇CO_2_ increase during early exercise due to transient carbon dioxide (CO_2_) uptake into body stores. With increasing work rate, a linear rise in heart rate (HR), oxygen pulse (V̇O_2_/HR) and ventilation (V̇E = minute ventilation) can be observed.

Exercise significantly improves ventilation/perfusion distribution (through increased pulmonary blood flow and deep breathing (increased tidal volume [V_T_]) resulting in an enlarged gas exchange area. This improved efficiency is reflected by a decrease in the ventilatory equivalents EqO_2_ (≈V̇E/V̇O_2_) and EqCO_2_ (≈V̇E/V̇CO_2_) because more oxygen (O_2_; V̇O_2_ ↑) is taken in and more carbon dioxide (CO_2_; V̇CO_2_↑) is eliminated relative to ventilation. The lowest point (nadir) of the ventilatory equivalents is where the lungs are working most effectively (e.g., only a small volume must be ventilated to breath in one litre of O_2_ or breath out one litre of CO_2_.

The partial pressures of O_2_ (P_ET_O_2_) and CO_2_ (P_ET_CO_2_) measured at the end of exhalation (end-tidal [ET]) correspond to the alveolar pressures, PAO_2_ and PACO_2_, in a healthy individual. P_ET_CO_2_ increases slightly and peaks during early exercise, reflecting the elevated CO_2_ production in exercising muscles, while increased peripheral O_2_ extraction (V̇O_2_ ↑) means that less O_2_ is exhaled (P_ET_O_2_ ↓).

**Aerobic-anaerobic transition zone**

As the exercise work rate continues to increase, ventilation increases (PAO_2_↑ and PACO_2_↓) without any more oxygen being taken up by the blood (no further increase in PaO_2_ or arterial O_2_ content) because haemoglobin is already fully saturated with oxygen. As a result of the maximally utilised aerobic metabolism, additional adenosine triphosphate (ATP) is generated via anaerobic glycolysis (advantage: rapid oxygen-independent energy supply; disadvantage: low energy yield: 2 mol of ATP for each 1 mol of glucose. The acidic end product of anaerobic glycolysis is lactate. The resulting hydrogen ions (H+) are buffered by sodium bicarbonate (HCO_3_^–^) to maintain a neutral pH: H^+^+HCO_3_^–^ → H_2_O and CO_2_. The resulting excessively produced CO_2_ stimulates a very strong ventilatory drive.

Due to this CO_2_-induced increase in ventilation, significantly more CO_2_ is exhaled (V̇CO_2_ ↑), while the increase in oxygen uptake (V̇O_2_↑) continues to rise only in parallel with the work rate. Accordingly, the increase in V̇CO_2_ is now significantly steeper than V̇O_2_ (anaerobic threshold [AT]). Since V̇E and V̇CO_2_ increase almost proportionally, the ratio V̇E/V̇CO_2_ ≈ EqCO_2_ remains relatively constant (or is minimally reduced), whereas the ratio of V̇E/V̇O_2_ ≈ EqO_2_ increases due to the relatively higher increase in V̇E versus V̇O2. The end-expiratory or alveolar O_2_ (P_ET_O_2_ [≈PAO_2_]) also increases as a result of CO_2_-mediated hyperventilation, whereas P_ET_CO_2_ (≈ PACO_2_) transitions into a plateau (or drops off slightly). The elevated O_2_ pulse tends to tail off with later exercise when the stroke volume cannot be further enhanced but the heart rate continues to rise linearly with increasing work.

Note: any pathophysiology that increases respiratory drive (e.g. dysfunctional breathing, PaCO_2_↑, pH↓) can cause or exacerbate dyspnoea. In hyperventilation, CO_2_ elimination exceeds CO_2_ production (washout of body CO_2_ stores); in hypoventilation, this is reversed.

**Anaerobic metabolism**

As exercise intensity continues to increase, more and more lactate accumulates in the muscles because the buffer base capacity for lactate-associated H^+^ is exhausted. The resulting metabolic lactic acidosis (pH↓) stimulates an additional strong central ventilatory drive (partial respiratory compensation of metabolic acidosis) beyond CO_2_-induced hyperventilation. As a result of the excessively increased ventilation, even more CO_2_ is exhaled (V̇CO_2_↑), while V̇O_2_ continues to increase only in parallel with the increasing work rate. Beyond the respiratory compensation point (RCP), V̇E increases at a greater rate than V̇CO_2_ (V̇E/V̇CO_2_↑), causing the P_ET_CO_2_ to decrease (increased ventilatory elimination of CO_2_). In addition, the respiratory exchange rate (RER: V̇CO_2_/V̇O_2_) increases disproportionately. The excessive increase in ventilation is associated with elevations of P_ET_O_2_ and of the two ventilatory equivalents EqO_2_ and EqCO_2_. In the anaerobic range, V̇O_2_ continues to tail off relative to heart rate, resulting in a flattened O_2_ pulse. Reaching the anaerobic range signals the impending termination of exercise.

**Recovery period (without Fig.)**

V̇E remains elevated for a short time (2–3 min) due to respiratory compensation of lactic acidosis with increased ventilatory elimination of CO_2_. In line with this, the ventilatory equivalents for O_2_ and CO_2_, RER (faster recovery of V̇O_2_ [↓] vs. V̇CO_2_ to baseline) and P_ET_O_2_ also increase before they rapidly return to normal.
